# Research and Application of Contactless Measurement of Transformer Winding Tilt Angle Based on Machine Vision

**DOI:** 10.3390/s23104755

**Published:** 2023-05-15

**Authors:** Jiazhong Xu, Shiyi Zheng, Kewei Sun, Pengfei Song

**Affiliations:** 1 School of Automation, Harbin University of Science and Technology, Harbin 150080, China; zsy99347@163.com (S.Z.);; 2 School of Mechanical Engineering, Harbin University of Science and Technology, Harbin 150080, China

**Keywords:** transformer coils, winding tilt angle detect, image self-segmentation and splicing, rotation projection method

## Abstract

In the process of producing winding coils for power transformers, it is necessary to detect the tilt angle of the winding, which is one of the important parameters that affects the physical performance indicators of the transformer. The current detection method is manual measurement using a contact angle ruler, which is not only time-consuming but also has large errors. To solve this problem, this paper adopts a contactless measurement method based on machine vision technology. Firstly, this method uses a camera to take pictures of the winding image and performs a 0° correction and preprocessing on the image, using the OTSU method for binarization. An image self-segmentation and splicing method is proposed to obtain a single-wire image and perform skeleton extraction. Secondly, this paper compares three angle detection methods: the improved interval rotation projection method, quadratic iterative least squares method, and Hough transform method and through experimental analysis, compares their accuracy and operating speed. The experimental results show that the Hough transform method has the fastest operating speed and can complete detection in an average of only 0.1 s, while the interval rotation projection method has the highest accuracy, with a maximum error of less than 0.15°. Finally, this paper designs and implements visualization detection software, which can replace manual detection work and has a high accuracy and operating speed.

## 1. Introduction

**Figure 1:**
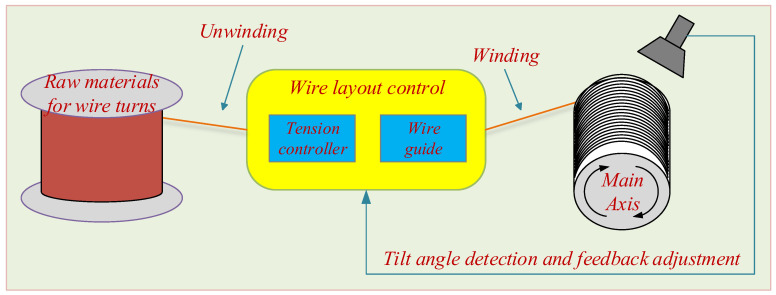
General process flow of producing coils.

The power system is the lifeline of modern industrial technology society, and transformers are the basic equipment for power transmission and distribution in the power system, widely used in power plants, converter stations, and substations. In the power industry, transformers occupy a very important position [1]. As a basic component of industrial power conversion systems, the stable performance of transformers is a key factor affecting the stability of distribution lines [2]. The main components of transmission and distribution transformers include iron core, winding, oil tank, insulation, and casing. The winding, which is wound on the iron core, is usually made of copper wire [3]. Currently, most winding coils are produced using automatic winding machines [4], and the production process [5] is represented in Figure 1.

**Figure 2:**
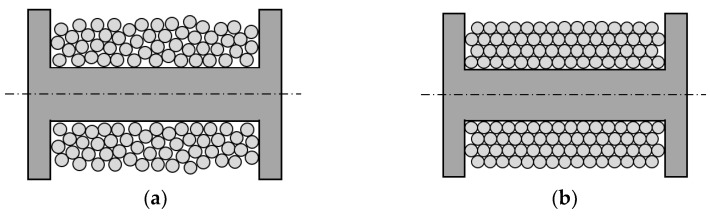
Winding types: (**a**) Irregular winding; (**b**) Regular winding.

During winding, the wires are wound into a linear array at a fixed initial tilt angle. The size of the initial tilt angle affects the number of turns that can be placed in the coil [6]. The typical goal is to wind as much wire as possible into the limited winding space [7]. During the winding process, the wire is in a tight state, and low mechanical accuracy and load mutations can easily cause changes in the winding tilt angle, which in turn causes the wire to slide in the current plane. This results in a chaotic ‘irregular winding’ (as shown in Figure 2a), with uneven adjacent and overlapping layers and many intersections and cavities in the winding structure, leading to a high potential difference and increasing the risk of voltage breakdown in the winding.

Conversely, winding with precise control of the tilt angle, referred to as ‘regular winding’ (as shown in Figure 2b), is compactly wound, and this orderly winding structure can meet higher quality requirements and achieve optimal use of the available winding space [8]. At the same time, the precise control of the winding structure ensures stable physical and electromagnetic characteristics, while the compact structure achieves higher mechanical stability, and the uniformly proportioned space gaps are more conducive to heat dissipation.

**Figure 3:**
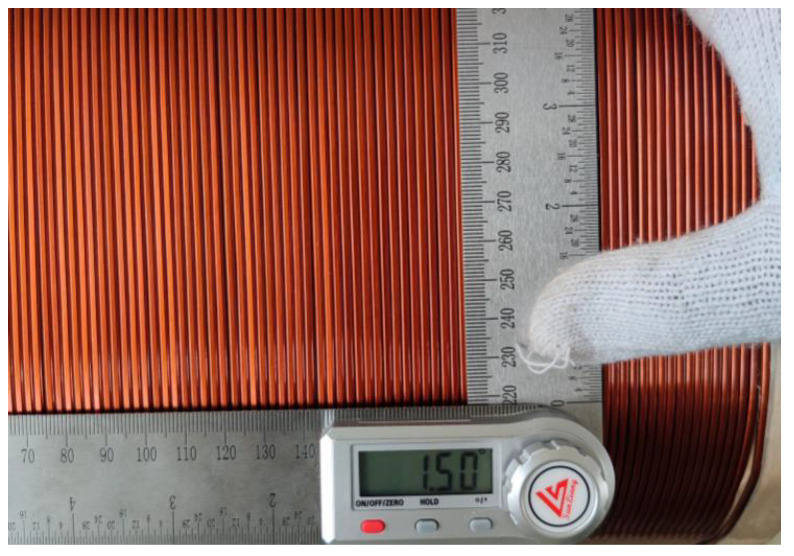
Manual measurement of winding tilt angle.

Therefore, during the winding process of the transformer coil, the tilt angle information of the winding between turns should be checked after each layer is wound. If the angle exceeds the specified range, the coil position must be manually adjusted. Currently, the factory uses manual visual inspection for each layer, as shown in Figure 3. However, the inspection time for this method is greater than 30 s and is prone to problems, such as eye fatigue, subjective dependence, and unclear inspection standards, making it difficult to accurately control product quality and there are often cases of misinspection.

In recent years, visual detection technology has achieved remarkable accomplishments in fields, such as image segmentation, image classification, object detection, image generation, etc. Compared with manual detection, it has the advantages of eliminating subjective interference and not requiring contact measurement [9,10], which can significantly improve the accuracy of the coil detection results. Although there are already studies showing that there are relatively few research achievements in the detection of winding coils, many scholars have conducted extensive research around angle detection in related fields.

Reference [11] conducted a study on the fiber-weaving angle in the production process of tubular-braided prefabricated components based on machine vision algorithms and compared the distribution of fibers in both spatial and frequency domains and developed a braiding angle detection algorithm using the phase rotation invariance of Fourier transform. The integrated system eliminates the need for manual measurement and improves the automation level of the braiding process. Reference [12] preprocessed the images of woven composite materials using lab transformation, block matching, and 3D filtering. Then, the authors obtained the grayscale edge map based on phase consistency and nonmaximum suppression. Finally, they calculated the surface braiding angle information through image rotation and gray projection. Reference [13] addressed the problem of slow angle detection of conductive slip ring brushes by applying machine vision and image processing algorithms to measure the key components of conductive slip ring brushes, using the Shi–Tomasi angular point detection algorithm to obtain the position information of the brushes and using the positioning block as a reference, the actual angle information of the brushes was calculated using tangent lines. Reference [14] investigated the optical properties of fiber materials and proposed a photometric stereo vision method to measure the diffuse reflectance and specular reflectance of each pixel as well as the fiber orientation. The root means square error of the fiber angle calculated was less than 1°. Reference [15] proposes a visual inspection method for measuring the diameter of enameled wires. The method employs Blob for image preprocessing and utilizes interpolation based on the Canny operator and the least squares method to measure the wire diameter. The absolute error is lower than 0.02 μm. Reference [16] employed the Canny edge detection algorithm as an image filter and overlayed the Fast Fourier Transform (FFT) transformation to provide feedback control on the fiber orientation during the carbon-fiber-reinforced plastic (CFRP) weaving process. The experimental results demonstrate that this method produces better filtering effects than the Sobel filter. Reference [17] presents a method for the precise winding of optical fibers that consist of a visual unit, the Sherlock visual recognition software, and an algorithm. This method employs image detection to identify the lag angle during the winding process as feedback for fiber alignment. The accuracy of the lag angle recognition for fiber alignment reaches ±0.1°. Reference [18] proposes a template-matching method that combines region matching and feature point detection to measure the relative angle between the line axis drive hole and the main spindle drive rod. This method offers advantages in terms of robustness, accuracy, and speed. Reference [19] investigated the impact of the fuel injection angle on diesel engine ignition and combustion in the cylinder. The study compared four methods for detecting the spray angle: a method based on the spray penetration length detection range, a method based on the fixed detection range of near-field and far-field spray regions, a method based on triangles, and a method based on the average of local data points. The results show that the different methods yield comparable spray angle values during steady-state operation but not during the initial injection. These findings guide selecting a suitable spray angle detection method.

Although the existing literature proposes detection methods that provide satisfactory results for the parameters under test, there are still issues regarding their applicability. Therefore, this article utilizes machine vision technology to design a noncontact method for measuring the tilt angle of transformer windings. Firstly, a 0° correction was performed, and image segmentation and splicing were used to obtain a single-wire image within the region of interest (ROI). The results of three angle detection methods were compared and discussed. The experimental results demonstrate that the method proposed in this paper requires less time compared to existing manual inspection techniques and is not affected by human errors. The remainder of the article is organized as follows: Section 2 introduces the preprocessing method for winding images, including the image segmentation and stitching method; Section 3 elaborates on the process of implementing the three winding angle detection methods in detail; Section 4 designs and implements a visualization detection software based on Python language and discusses the results of the angle detection experiments.

## 2. Methods Design

**Figure 4:**
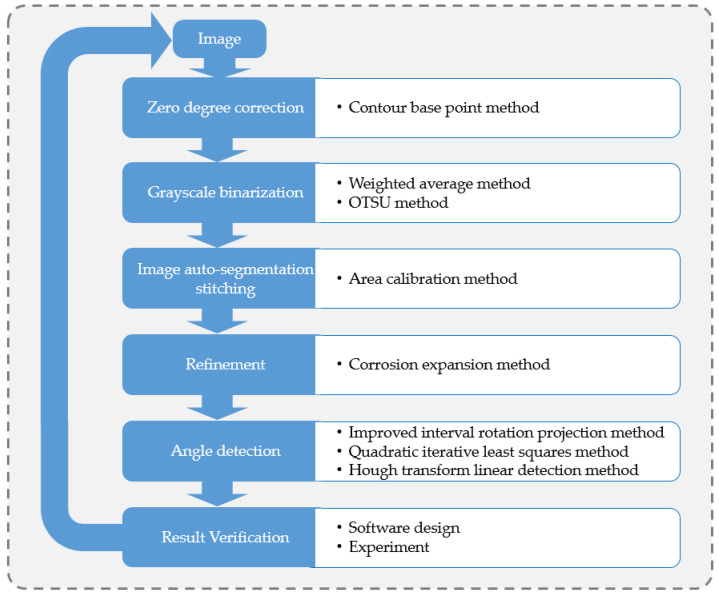
The design process for the detection program.

To develop a suitable machine vision system, the following scheme implementation process was designed as shown in Figure 4, and the entire detection process can be divided into two main stages, namely image preprocessing and postprocessing. During image preprocessing, 0° correction and grayscale conversion and a self-segmentation and splicing method were used to obtain the skeleton image of each wire. Then, using the visualization detection platform, the information related to the winding angle was obtained using three different angle detection methods.

## 3. Image Preprocessing

### 3.1. 0° Correction

**Figure 5:**
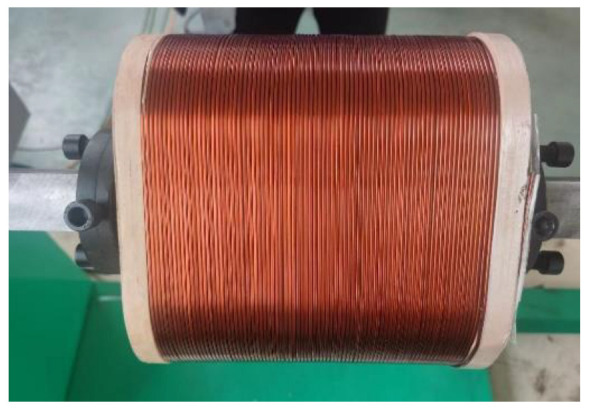
Structure of coils.

During the production process, we obtained images of the winding coils as shown in Figure 5. The coils had a roughly three-section structure, including the insulation material on both sides and the wires in the middle. Before detecting the winding tilt angle, the acquired image needed to be zero-corrected to obtain a reference parameter for angle detection.

**Figure 6:**
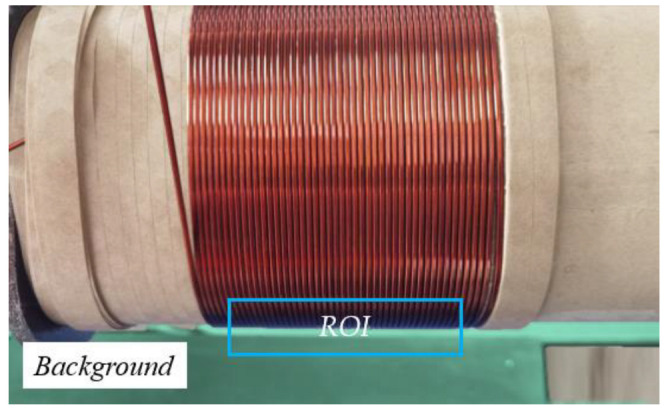
The base point method measures the reference angle.

We observed that there was a clear demarcation line between the foreground and background of the winding, so this paper used the ROI of the intersection of the foreground and background to detect the demarcation pixel coordinates, marked in the blue rectangular box in Figure 6 and fitted a series of coordinates to obtain the overall angle of the coil, which was subsequently used as the detection benchmark and then the winding tilt angle of the winding.

### 3.2. Detection of Location Segmentation

**Figure 7:**
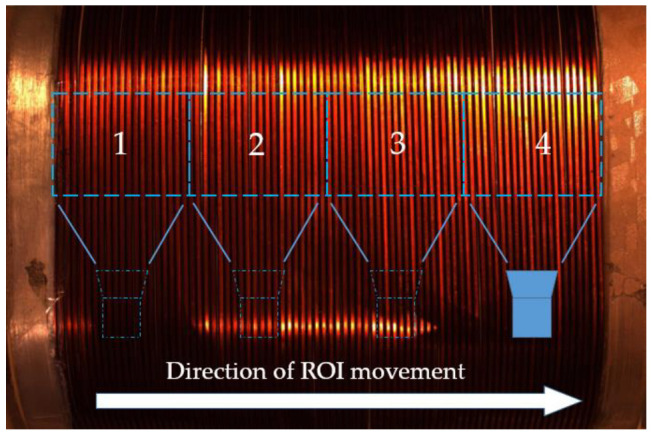
The direction of camera movement, the numbers in the figure indicate the detection order of ROI.

Figure 5 and Figure 6 show that when the shooting distance of the lens is increased, the circular structure of the winding will cause a visual phenomenon similar to barrel distortion on the wires on both sides of the image, which cannot be used as a sample image for detection. Therefore, in this article, we adopted the approach of zooming in the lens and taking multiple shots to obtain the ROI, as shown in Figure 7. The winding was segmented into several small sections, and each section was detected separately to ensure that the results were not affected by pseudodistortion.

### 3.3. Grayscale Binarization

**Figure 8:**
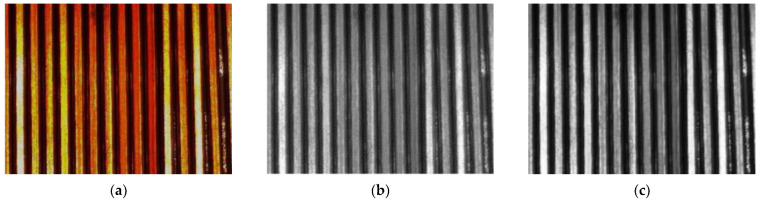
Image graying: (**a**) Sample images; (**b**) Average method; (**c**) Weighted average method.

Color ROI images contain three-channel data, which often leads to the issue of excessive data volume when processing such images [20]. To minimize data loss while reducing the volume, multichannel images can be converted to single-channel images. Currently, there are two common methods used for this purpose: the average method [21] (Gray = (R + G + B)/3) and the weighted average method that considers the perceptual differences of human eyes to different brightness [22] (Gray = 0.299 × R + 0.587 × G + 0.114 × B). As shown in Figure 8, (b) uses the average method to convert (a) into a grayscale image; (c) uses a weighted average method to convert (a) into a grayscale image.

To accurately segment each wire from the ROI, binary thresholding was performed on the grayscale image. The OTSU method [23] was utilized for binary thresholding in this paper.

**Figure 9:**
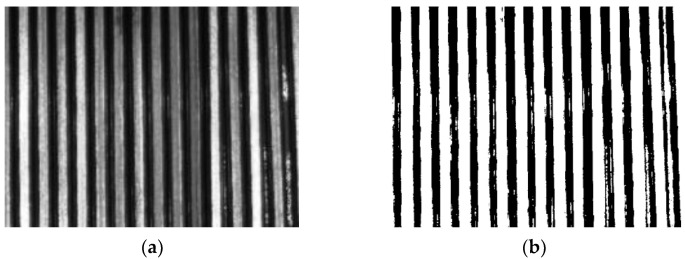
Image binarization using the OTSU method: (**a**) grayscale image; (**b**) binarized image.

The OTSU binarization method can be outlined as the following steps:

1. Calculate the histogram of the grayscale image and calculate the number of pixels occupied by each pixel value from 0 to 255.
2. Iterate through the threshold values 0–255, with pixels less than or equal to the threshold value being the background and pixels greater than the threshold value being the foreground.
3. Calculate the ratio of the number of background pixels to the total number of pixels and the average value of the background pixels.
4. Calculate the proportion of the number of foreground pixels to the total number of pixels and the average value of the foreground pixels.
5. Calculate the interclass variance or intraclass variance, when the threshold that maximizes the interclass variance or minimizes the intraclass variance is the optimal threshold.
6. Binarize the image using the best threshold. After the binarization process, the binarized image obtained from the grayscale image is shown in Figure 9b.

### 3.4. Image Self-Segmentation and Splicing

In binary images, white areas represent ideal wire connections, while black areas represent wire gaps and insulation layers. However, in grayscale images, due to changes in the winding angle of wires within the ROI area, the insulating adhesive layer hidden underneath the wires is exposed, creating a light reflection on the wire surface that appears as the same grayscale value as the wire itself, resulting in a misidentification of the wire. Therefore, an image self-segmentation and stitching method is proposed to effectively eliminate this interference.

**Figure 10:**
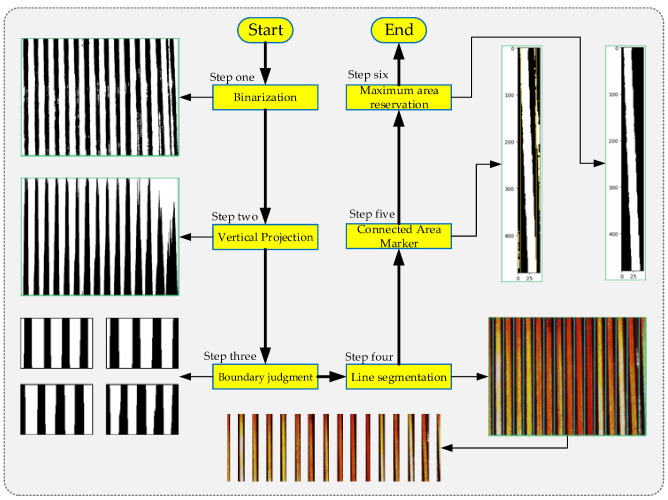
Image self-segmentation splicing method.

An image self-segmentation splicing method can achieve the accurate segmentation of the wire and the processing of the separated wire to eliminate the interference information outside the wire, and the implementation flow of the method is shown in Figure 10.

**Figure 11:**
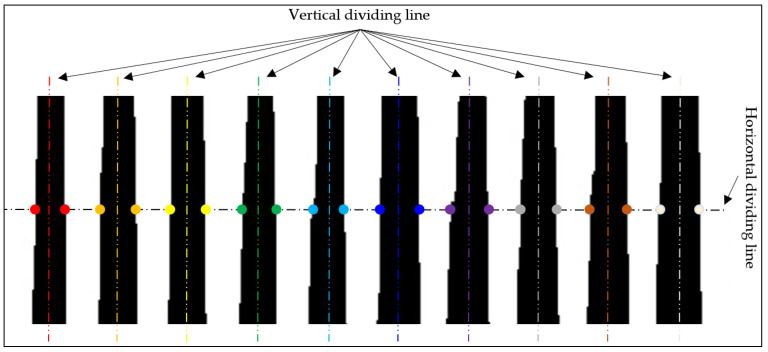
Projection image splitting.

After binary thresholding the ROI, a vertical projection operation was performed (corresponding to Step two in Figure 10). The resulting projection image shows black and white striped patterns, where the black stripes represent the gaps between the wire connections. Our goal was to separate adjacent wires. The steps for wire separation were as follows: first, a horizontal dividing line was determined, and then the pixels were traversed from one side of the ROI to the other side starting from the height of the dividing line. When a color transition occurs, the pixel coordinates at the transition were recorded. After obtaining all the coordinates, they were classified based on their position, and the pixels on both sides of the black stripes were grouped together, as shown in the figure by pixels of the same color. In this example, there was a total of 10 groups of pixels. Then, the column of the middle pixel in each group was calculated, which was located within the black projection pixels and served as the dividing line for the wires. Using this method, 10 dividing lines were determined, as shown in Figure 11.

Additionally, the selection position of the ROI was not fixed. Its starting position can begin from the wire side or from the gap side, and the ending position can also be on either side. Therefore, based on the distribution on both sides of the ROI, four situations can be formed. These correspond to Step three shown in Figure 10. For the convenience of distinction, we defined these four types as (a) White–Black–Black–White (WBBW), (b) White–Black–White–Black (WBWB), (c) Black–White–Black–White (BWBW), and (d) Black–White–White–Black (BWWB).

**Figure 12:**
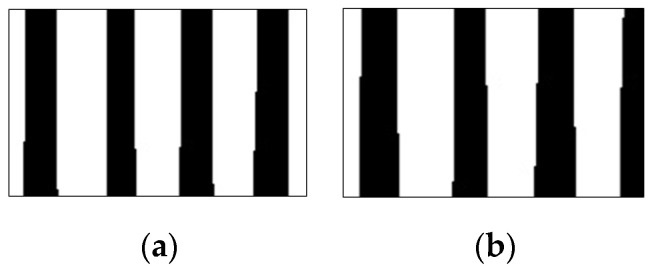

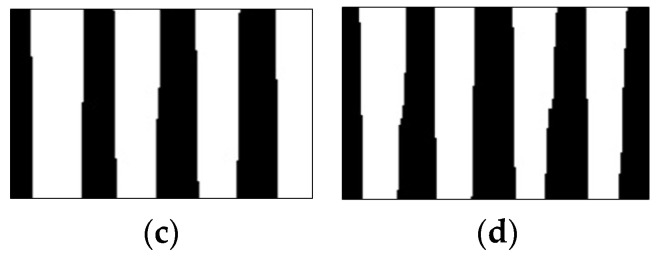
Lead-splitting models customized to different ROI projections: (**a**) white–black–black–white; (**b**) white–black–white–black; (**c**) black–white–black–white; (**d**) black–white–white–black.

**Figure 13:**
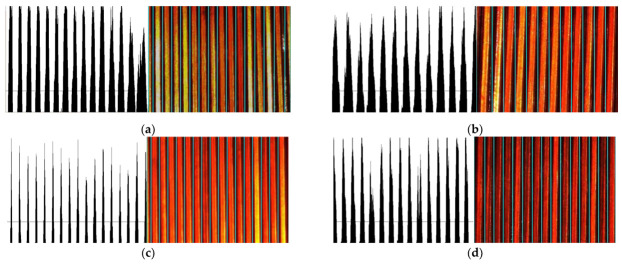
Examples correspond to the four split types in Figure 12: (**a**) segmentation result of WBBW-type; (**b**) segmentation result of WBWB-type; (**c**) segmentation result of BWBW-type; (**d**) segmentation result of BWWB-type.

After Step two and Step three operations, the coordinates of the split wires were obtained, and then each wire was split out individually. The splitting results of the four types of ROI in Figure 12 are shown in Figure 13.

**Figure 14:**
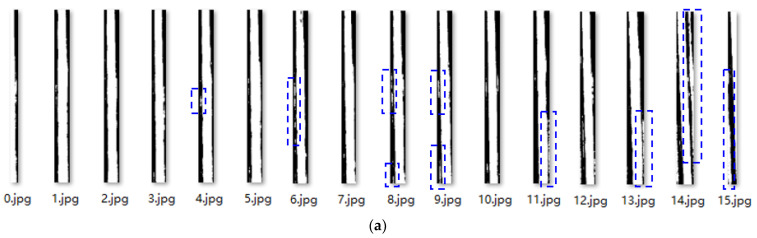

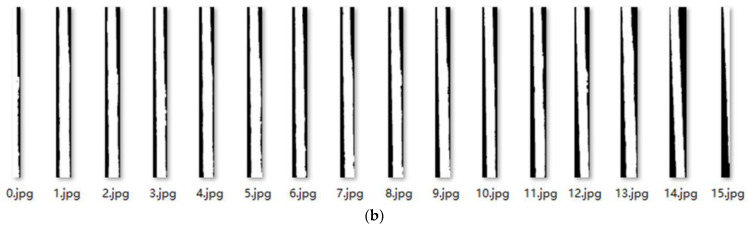
(**a**) Splitting results of Figure 9b; (**b**) Results after area calibration method processing, interference pixels are marked in the rectangular box.

Figure 14 shows the results of the segmentation of the binary images of the wires. Upon observing each image, it is evident that there are many interference points caused by the reflection of the insulating layer adhesive, and in addition to the wire body, these points can easily affect the results of skeleton extraction. For example, in 14.jpg, this paper proposes a calibration method based on the principle of image connectivity to remove interference.

**Figure 15:**
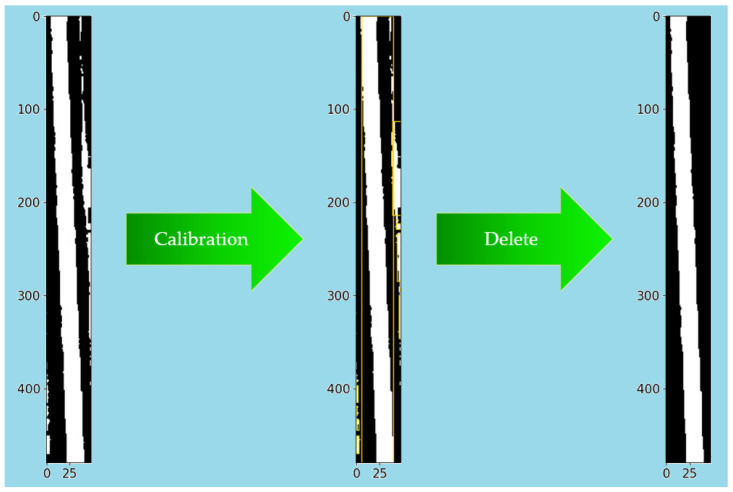
Area calibration method.

Image contour refers to the curve that connects all continuous points with the same color or intensity along the boundary. It is a useful tool for shape analysis, object detection, and recognition [24]. The proposed calibration interference removal method in this paper mainly consisted of the following five steps: (1) determination of connected regions; (2) region labeling; (3) contour calibration; (4) area sorting; and (5) label deletion. This process can be represented by Figure 15, corresponding to Step five and Step six in Figure 10.

**Figure 16:**
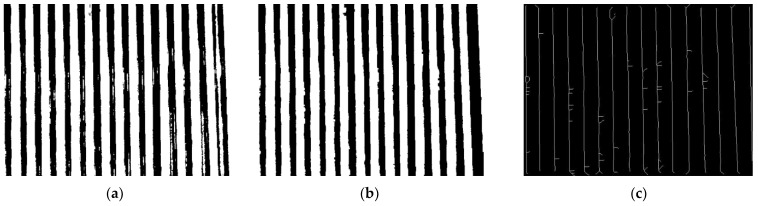
(**a**) Figure 14a recombination; (**b**) Figure 14b recombination; (**c**) Skeleton image.

After removing the interference using the calibration deinterference method, a binarized image with only the wire information in the figure was obtained. After all segmented wires were processed, the results in Figure 14b were obtained, and the wire images were recombined to obtain the results in Figure 16b.

### 3.5. Skeleton Extraction

In each of the binary images obtained after removing noise, only the information of a single wire at the same position as the original image was retained. However, to achieve a faster processing speed and smaller memory usage, a more compact image representation, i.e., skeleton [25], was sometimes used. The skeleton is a structure that removes all redundant pixels while retaining the main shape [26]. In reference [27], the skeleton of an image can be obtained through erosion and dilation operations. Based on this principle, this paper designed a set of wire skeleton extraction processes, which are as follows:

1. First, use the etching operation on the image; each time the etching becomes narrower and thinner.
2. Perform image open operation processing; some pixels of the image will be deleted, and these deleted pixels are part of the skeleton.
3. Add the deleted pixels to the skeleton map.
4. When the sample image is eroded to no pixels, end the iteration, and finally obtain the skeleton image of the previous step.

The result after skeleton extraction is shown in Figure 16c.

## 4. Detection Method

To make an accurate measurement of the winding angle of the winding coil, three methods for detecting the angle were investigated in this chapter: an improved interval rotation projection method, a quadratic iterative least squares method, and a Hough transform method.

### 4.1. Improved Interval Rotation Projection Method

The detection principle of the rotation projection method is as follows: the skeleton of a wire can be regarded as a combination of a series of single pixels arranged in a certain direction in the plane. Assuming that a skeleton curve with 1000 pixels is exactly parallel to the *Y*-axis, a large peak will appear at the highest point of the projection when projecting in the *X*-axis direction [28]. If the skeleton is not parallel to the *Y*-axis, the peak value in the projection image will be smaller.

**Figure 17:**
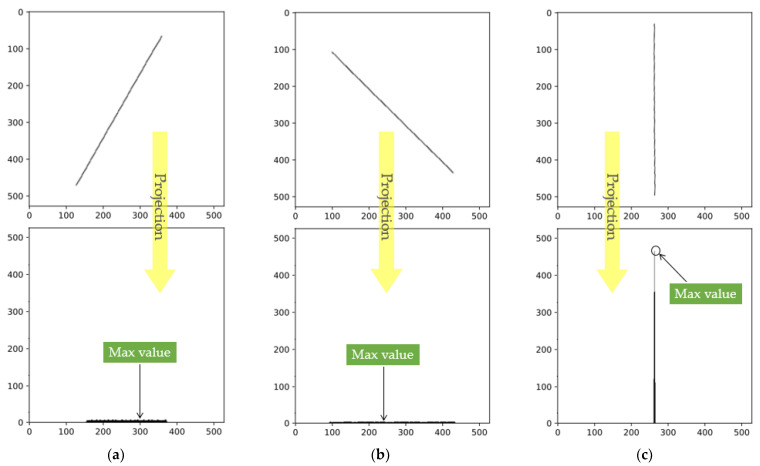
The projection peaks correspond to different angles: (**a**) Projection at 30°; (**b**) Projection at −45°; (**c**) Projection at 2°.

Based on the above projection characteristics, after obtaining a single-wire skeleton image, the image was first filled to a square shape, and the original image angle was set to 0° with a detection accuracy of 0.1°. Starting from 0°, a projection was made every 0.1° counterclockwise, and the maximum peak value in each projection was calculated. Figure 17 shows the rotated images and projection intensities at rotation angles of 30°, −45°, and 2°.

**Figure 18:**
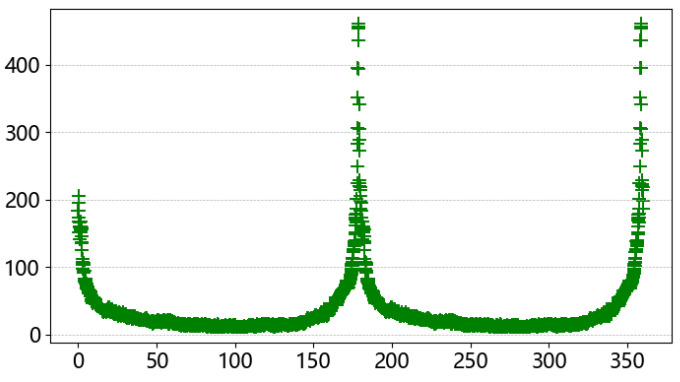
Pixel component intensity map.

When rotated 360°, the projection peak in each projection was extracted separately, totaling 3600 coordinate points, and then the angle was used as the horizontal coordinate and the peak point as the vertical coordinate to obtain the peak intensity map of the projection angle shown in Figure 18.

**Figure 19:**
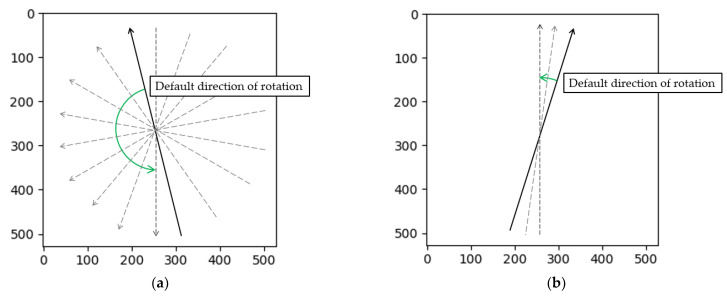
(**a**) The left-tilt-type obtains the angle of rotation at maximum projection; (**b**) The right-tilt-type obtains the angle of rotation at maximum projection.

In the 360° rotation pixel intensity map, there are two peaks that correspond to the results of the two projection peaks when the skeleton is vertical. These two peaks appear at 178.2° and 358.2°. However, this angle is not the true inclination angle of the wire, and a further angle conversion was required to obtain the true winding angle. Due to the special design of the coil-winding process, the winding angles of adjacent wire layers are relative. As shown in Figure 13a, there is a leftward trend, while in Figure 13b, there is a rightward trend. However, the default rotation projection direction in the algorithm is counterclockwise, as shown in Figure 19. This means that wires that lean to the left need to rotate almost half a circle to produce peak data, while wires that lean to the right only need to rotate a small angle to produce peak data. Therefore, the peak data obtained from these two types of tilted wires will differ significantly. The purpose of the conversion was to reconstruct these two peak data and reflect the true winding angle information.

**Figure 20:**
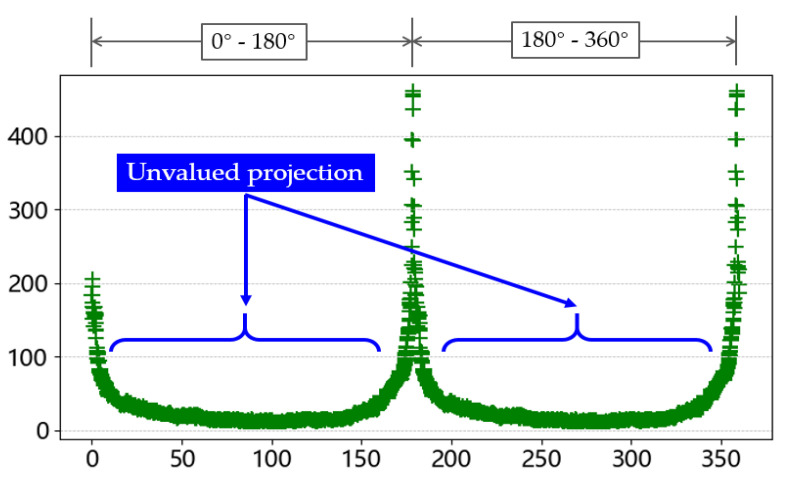
Most of unvalued projection.

During the testing phase, it was found that processing a single image required an average of 2 min and 23 s, with an average of 15 wires per region of interest. This resulted in processing all data taking too long to meet real-time requirements, as most of the time was spent on rotating projections. However, in actual production, the range of wire-winding angles is very small. An analysis of Figure 20 shows that the results of the projections in the 0–180° and 181–360° ranges are the same. Moreover, many projections produced beyond a certain angle have no practical value. Therefore, this paper proposes the following simplification methods: (1) reduce the range from 360° to 180°; (2) determine the direction of wire inclination before performing the projection, and if the wire tilts to the left, perform clockwise direction projection; if the wire tilts to the right, perform counterclockwise direction projection; and (3) limit the rotation interval to within 10°, while still using a rotation step size of 0.1°.

**Figure 21:**
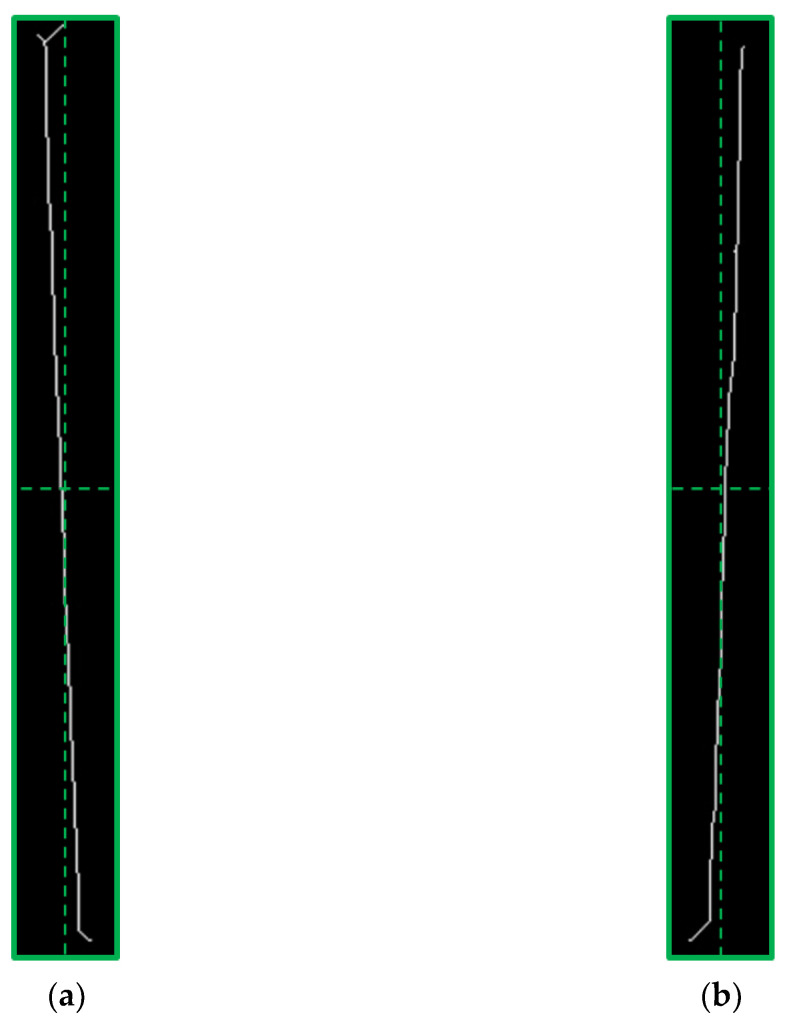
(**a**) Left-leaning, (**b**) Right-leaning.

In addition, the method to determine the tilt angle mentioned here was as follows: first, the image was evenly divided into four regions, and then the number of white pixels in each of the four regions was calculated separately. Next, the pixel counts of the two regions on the diagonal were added together because the skeleton only existed in the regions along the diagonal as shown in Figure 21. Therefore, by summing up the pixel counts of all four diagonal regions, the initial tilt direction of the skeleton could be determined.

**Figure 22:**
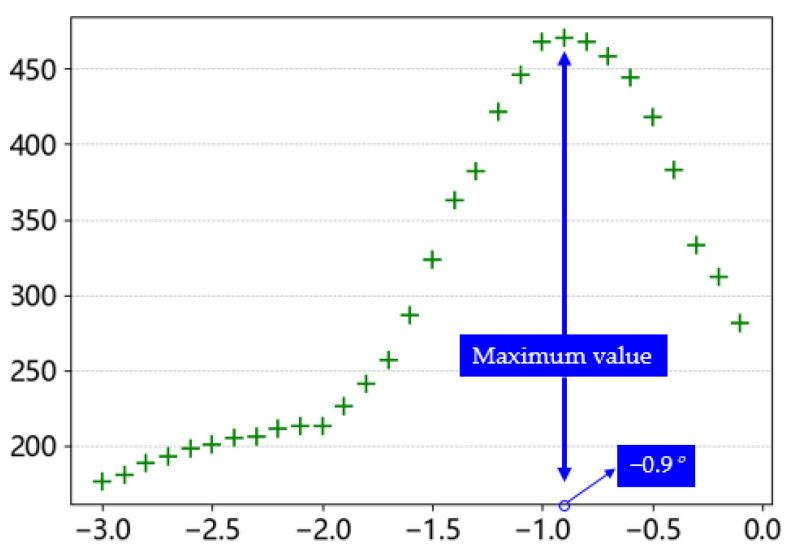
Optimized pixel component intensity map.

The peak intensity map and the winding angle of all wires within the ROI obtained by the improved algorithm are shown in Figure 22. The real-time performance of the improved algorithm was greatly improved, and the detection time of a single wire was reduced to 15 s, which greatly optimized the detection time.

### 4.2. Quadratic Iterative Least Squares

Ordinary least squares (OLS) is the most-used linear regression method, which seeks the best function fit for data by minimizing the sum of squared errors [29]. As previously mentioned, the wireframe is composed of many individual pixel points, which are fixed in their positions in the image. We can view the image as a two-dimensional plane and seek a line of best fit through the wireframe points by using the OLS method, minimizing the sum of the vertical distances between the points and the line. Therefore, the fitted result can represent the position and direction of the wireframe line on the plane.

Assuming that the set of coordinates constituting the skeleton image is: [*x_i_, y_i_*] and the fitted straight line is denoted as *y* = *kx* + *b*, we defined the sum of squares of the fitted deviations as:

(1) $$\sum_{i=1}^{N}{\left[y_{i}-(kx_{i}+b)\right]}^{2}$$

Find the partial derivative for *k*, *b*:

(2) $$\frac{\partial }{\partial k}\sum_{i=1}^{N}{\left[y_{i}-(kx_{i}+b)\right]}^{2}=-2\sum_{i=1}^{N}[y_{i}-(kx_{i}+b)]x_{i}=0$$

(3) $$\frac{\partial }{\partial b}\sum_{i=1}^{N}{\left[y_{i}-(kx_{i}+b)\right]}^{2}=-2\sum_{i=1}^{N}[y_{i}-(kx_{i}+b)]=0$$

Simplify (2), (3):

(4) $$k\sum_{i=1}^{N}x_{i}{}^{2}+b\sum_{i=1}^{N}x_{i}=\sum_{i=1}^{N}x_{i}y_{i}$$

(5) $$k\sum_{i=1}^{N}x_{i}+bN=\sum_{i=1}^{N}y_{i}$$

Derive the specific values of *k*, *b*:

(6) $$k=\frac{N(\sum_{i=1}^{N}x_{i}y_{i})-(\sum_{i=1}^{N}x_{i})(\sum_{i=1}^{N}y_{i})}{N(\sum_{i=1}^{N}x_{i}{}^{2})-{\left(\sum_{i=1}^{N}x_{i}\right)}^{2}}$$

(7) $$b=\frac{\left(\sum_{i=1}^{N}x_{i}{}^{2})(\sum_{i=1}^{N}y_{i})-(\sum_{i=1}^{N}x_{i})(\sum_{i=1}^{N}x_{i}y_{i}\right)}{N(\sum_{i=1}^{N}x_{i}{}^{2})-{\left(\sum_{i=1}^{N}x_{i}\right)}^{2}}$$

**Figure 23:**
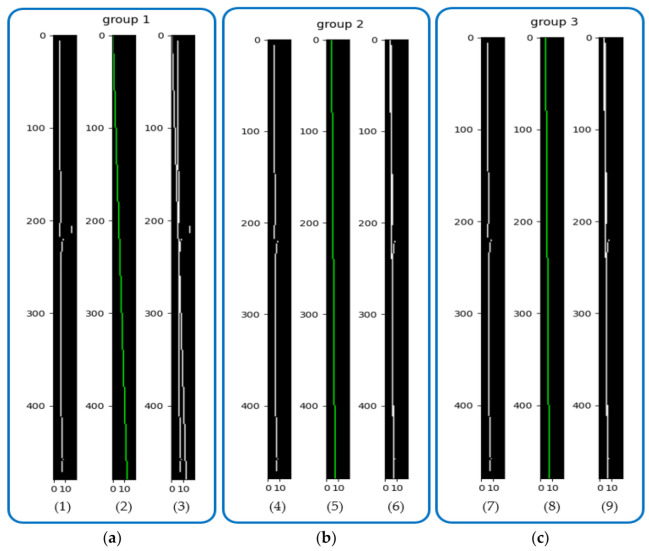
QILS fit, where the first image of each group is the skeleton image, the second image is the result of the fit, and the third image is the comparison of the results: (**a**) OLS fit; (**b**) Second iteration fit; (**c**) Third iteration fit.

However, the least squares method also has significant limitations. Because this method used all data points to calculate the result, the presence of outliers can seriously affect the accuracy of the fitting result. For example, in the two wireframe images of the skeleton shown in Figure 21, there are spikes at the two ends of the skeleton. Similarly, there are some outliers in Figure 23a. To address the interference of these outliers, a method called iterative reweighted least squares (IRLS) is proposed in reference [30]. This method assigns a weight to each target point and performs multiple iterations. In each iteration, the method adjusts the weight of each target point by reducing the weight of the error point among all target points, thus eliminating the influence of outliers. As the number of iterations increases, the predicted results become closer to the actual results, but correspondingly, the computation time also increases.

To meet the real-time requirements of the algorithm, this paper was inspired by IRLS and proposes a quadratic iterative least squares (QILS) method. The algorithm consists of the following steps:

1. Perform a least squares fit on the initial data points to obtain the first fitting result;
2. Calculate the vertical distance of all data points to the first fitting result;
3. Set a threshold for the vertical distance and exclude the data points whose distance exceeds the threshold;
4. Perform another least squares fit on the remaining points to obtain the second fitting result.

Figure 23 (1)–(3) show the first fitting results; Figure 23 (4)–(6) show the second iteration results; and Figure 23 (7)–(9) show the third iteration results. In the first set of results, Figure 23 (3) shows the comparison between the fitted and actual results, and there is a large angular deviation between them. After the second iteration, the fitted and actual results in Figure 23 (6) are already very close to each other. After the third iteration, the results do not change significantly, so this paper used iteration two that met the requirements.

### 4.3. Hough Transform Linear Detection Method

The Hough transform is a method used in image analysis to detect basic geometric shapes, such as lines, circles, and ellipses. This method was initially proposed by Paul Hough [31] in 1962 and later extensively used by Richard Duda and Peter Hart [32] in 1972. The Hough transform for lines assumes a one-to-one correspondence between lines in the image space and lines in the parameter space. For instance, the equation of a line passing through any point A (*x*_0_, *y*_0_) in the image space can be represented as *y*_0_ = *kx*_0_ + *b*, where *k* is the slope and *b* is the y-intercept. When this line is transformed to the parameter space, the resulting equation is *b* = −*kx*_0_ + *y*_0_. If multiple points are present in the image space, collinear points in the parameter space will yield a unique set of (*k*, *b*) data, which, when transformed back to the image space, provide a means of determining collinear lines. However, if the line in the image space is perpendicular to the *X*-axis and the slope *k* is undefined, it cannot be represented in the parameter space. Therefore, polar coordinates are typically used as the parameter space. Equation (1) expresses the formula for conversion from image space to polar coordinate space.

(8) r=xcosθ+ysinθ

**Figure 24:**
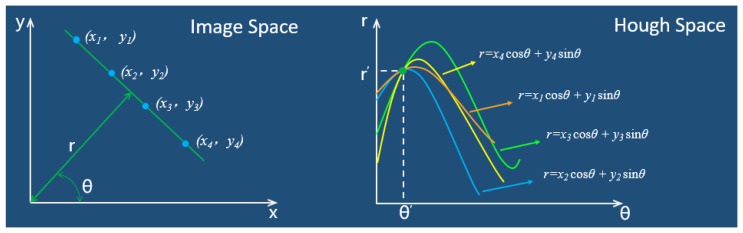
The transformation process from image space to polar parameter space.

**Figure 25:**
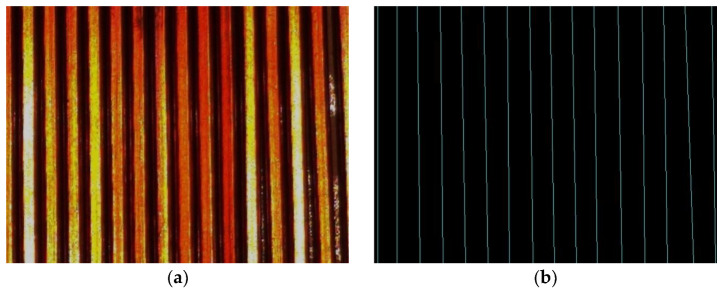
Probabilistic Hough transform detection results: (**a**) Sample images; (**b**) Detection results.

From the above, each point (*r*, *θ*) in the parameter space corresponds to a straight line in image space, or, in other words, a point in image space corresponds to a curve in the parameter space. As shown in Figure 24, the transformation process from image space to polar coordinate parameter space is as follows:

In this paper, the probabilistic Hough transform algorithm was used to detect the winding angle. The probabilistic Hough transform principle is simple, requires small memory usage, and can obtain the endpoint coordinates of the line segments. The main steps are as follows:

1. Randomly select foreground points in the edge image and map them to a polar coordinate system to draw curves.
2. When the curves intersect in the polar coordinate system and reach the minimum threshold, find the position of the intersection in the image space.
3. Search for points on the edge image that are on this line, connect them to form line segments, and record the starting and ending coordinates.
4. Repeat steps 1–3, and the final fitted result is shown in Figure 25.

## 5. Experiment and Discussion

### 5.1. Software Design and Hardware Construction

**Figure 26:**
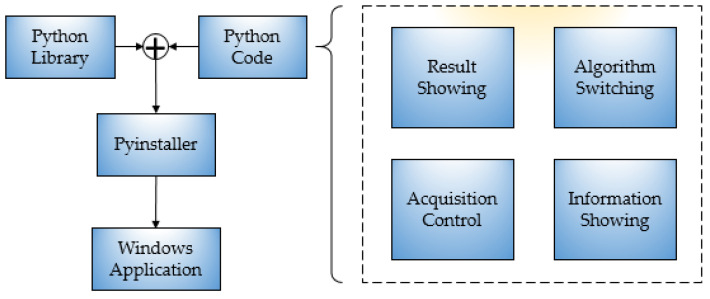
System framework design block diagram.

**Figure 27:**
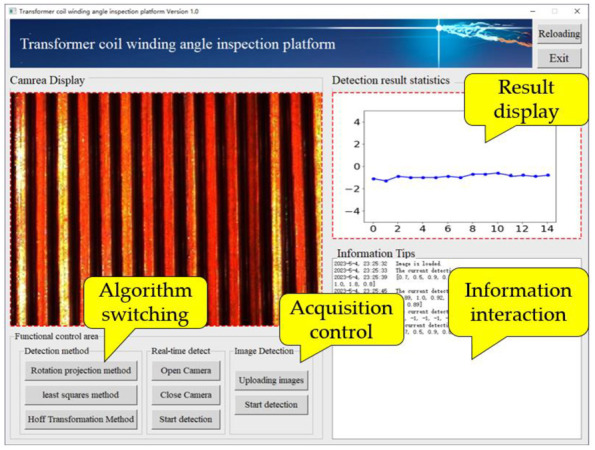
Detection software.

The algorithm presented in this paper was written and implemented in Python, and a visual detection platform was designed in conjunction with PyQt5, which was packaged into an executable program for Windows through the Pyinstaller tool. The flow is shown in Figure 26. The detection algorithm can be applied to actual production through this platform. The main functions of the platform included camera calling, image processing, angle measurement, and information exchange. The platform interface is shown in Figure 27, and the front end includes image acquisition control, detection algorithm switching, image display, and results display. Image detection supports static and dynamic methods. Static detection can be achieved by uploading local sample images, while dynamic detection can be performed by connecting an industrial camera for real-time image detection.

**Figure 28:**
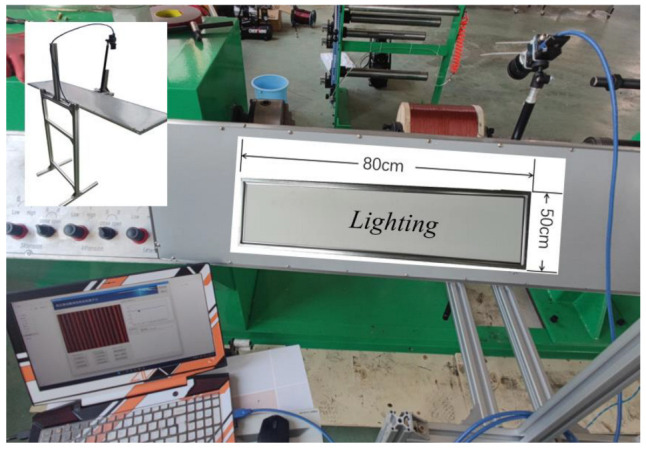
Winding equipment and image capture device.

Table 1 shows some of the parameters of the external industrial camera used in this paper, while the structure of the image acquisition device is shown in Figure 28. We designed an image acquisition device similar to the one in reference [33], which fixed the camera on an adjustable shooting frame to avoid direct contact with the main equipment and effectively reduce the interference caused by equipment vibration.

### 5.2. Single Conductor Winding Tilt Angle Detection Experiment

To provide a more intuitive demonstration of the advantages and disadvantages of the three detection methods, we designed two sets of detection experiments. The first set adopted a static detection method, selecting six images of a single wire from the local database and comparing the detection results and detection time with the manual detection results. Table 2 presents the detection data.

**Figure 29:**
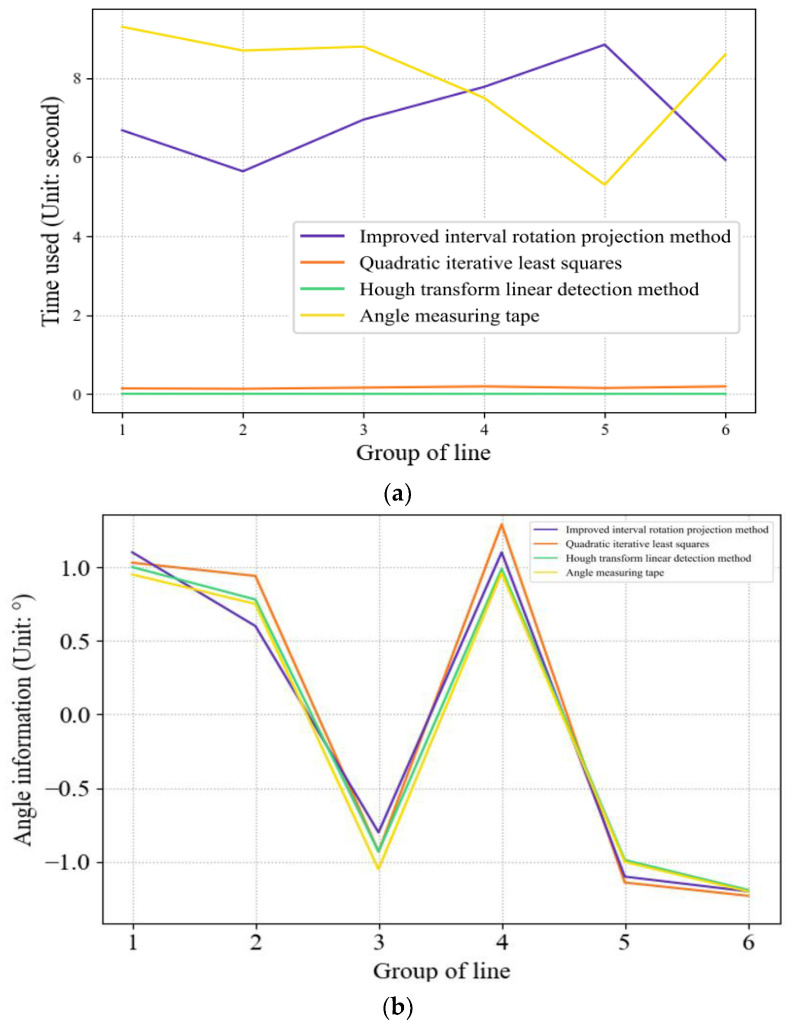
Comparison of the time consumed and test results by the four measurement methods: (**a**) Time consumed; (**b**) Test result.

Comparing the static single conductor tilt angle detection results in Table 2, all three detection methods described in the paper can correctly detect the true angle, and combined with the time consumption data of each algorithm shown in Figure 29, we concluded the following: among the 6 groups of samples, the maximum error between the improved rotational projection method and the manual measurement is 0.0°, which is the smallest among the three methods, and the results of the remaining groups are also the closest to the manual measurement results. The results of the remaining groups are also the closest to the manual measurement results, but the single detection time is around 20 s. The analysis may be because the logic processing of the algorithm did not achieve optimal processing. The second iterative least squares and the Hough linear detection algorithm are both below 0.5 s in time processing, and only the second iterative least squares of the third group of 6 samples has the problem of large results because there are still some outliers in this group of samples before the second iteration, which interferes with the fitting results. The Hough transform method, as the fastest detection algorithm, showed optimal results both compared to the previous two algorithms and compared with manual detection. All the above conclusions achieved the expected objectives.

### 5.3. Multiconductor Winding Tilt Angle Detection Experiment

**Figure 30:**
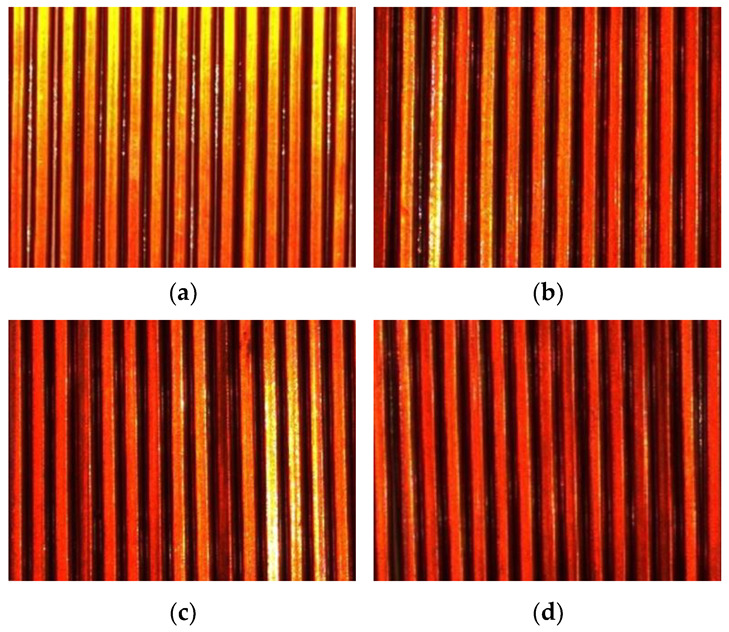
Images of the acquired multiconductor samples: (**a**) Sample image 1; (**b**) Sample image 2; (**c**) Sample image 3; (**d**) Sample image 4.

**Figure 31:**
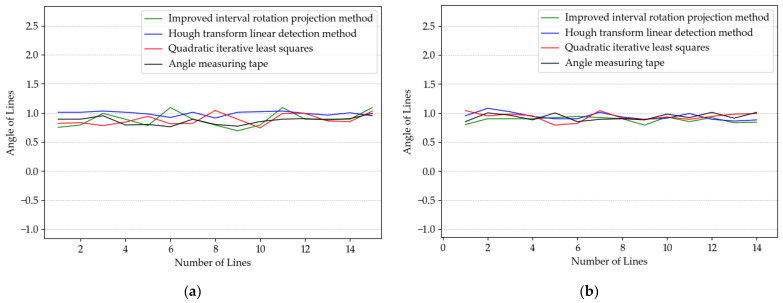

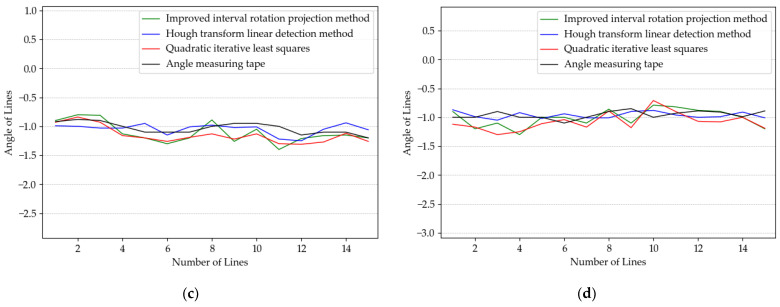
Multiconductor image detection results. (**a**) Result of Figure 30a. (**b**) Result of Figure 30b. (**c**) Result of Figure 30c. (**d**) Result of Figure 30d.

The second group of experiments used the dynamic detection method, where the camera was controlled by software to take real-time winding images, and four ROI images were intercepted after zero correction, as shown in Figure 30. In this part of the experiment, we tested each image with the same three image detection algorithms and compared the results with the manual measurements, because the number of wires within different ROIs may not be the same, so there will be deviations in the number of results obtained. Figure 31 shows the specific angular values of the individual wires within each ROI in Figure 30.

From the results of the multiwire image inspection, the results of all three inspection methods and manual measurement do not show large errors, except for the improved interval rotation projection method, which has a relatively long inspection time, and the quadratic iterative least squares method and the Hough transform linear detection method both show more efficient results than manual measurement and can be changed according to the usage requirements in actual production. Therefore, the experimental comparison proves that the detection method in this paper has the value of a practical application.

## 6. Conclusions

This article presents a contactless measurement method based on machine vision technology for detecting the inclination angle of the winding coil of a power transformer during production. The method used cameras to capture images of the winding coil and processed the images to design and implement a visualization detection software that can replace manual detection work and has high accuracy and running speed.

Firstly, we discussed the problems associated with traditional visual detection methods using human eyes, such as being time-consuming, producing large errors, being prone to eye fatigue, relying on subjective awareness, and lacking clear detection standards. To address these issues, we adopted a contactless measurement method based on machine vision technology.

Secondly, in the image preprocessing stage, we proposed an image self-segmentation and splicing method that used vertical projection to determine the boundary determination conditions of the wire’s binary image, performed ROI segmentation, and used calibration methods to remove interference from reflection and varnish. Finally, the images were combined and the skeleton is refined.

To measure the winding inclination angle, we compared and analyzed three angle detection methods: the improved interval rotation projection method, the optimized least squares method, and the Hough transform line detection method. The experimental results show that the measures proposed for improving the interval rotation projection method can improve its real-time performance, and the measures added to the least squares method for the Hough transform showed good follow-up ability.

Finally, based on the characteristics of the wire-winding coil, we built an image acquisition system and designed an algorithm visualization detection platform with main functions including image acquisition control, detection algorithm switching, image display, and results display.

Compared with the manual measurement method, the machine vision technology used for parameter measurement saves human resources to a certain extent, while ensuring the accuracy of the measurement results, which is not available in the existing manual inspection methods, but there is still a lack of details in the image processing; as a topic for future research, we will consider combining deep learning algorithms on this basis to achieve more accurate measurement results.

## Figures and Tables

**Table 1:** Parameter of camera and lens.

Physical Appearance	Parameter	
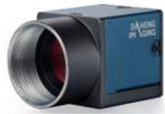	Industrial Camera	
	Model	MER2-1220-32U3C
	Pixel resolution	4024 × 3036
	Pixel size	1.85 μm × 1.85 μm
	Frame rate	32.3 fps
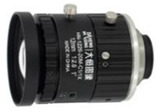	Lens	
	Model	HN-1226-20M-C1/1X
	Focal length	12 mm
	Maximum supported pixels	20 million
	Distortion factor	0.0

**Table 2:** Angle detection experimental data.

	Improved Interval Rotation Projection Method		Quadratic Iterative Least Squares Method		Hough Transform Linear Detection Method		Angle Measuring Tape	
	Angle (*°*)	Time (s)	Angle (*°*)	Time (s)	Angle (*°*)	Time (s)	Angle (*°*)	Time (s)
Group 1	1.1	6.68	1.03	0.14	1.00	0.003	0.95	9.30
Group 2	0.6	5.64	0.94	0.13	0.78	0.003	0.75	8.70
Group 3	−0.8	6.95	−0.93	0.16	−0.93	0.002	−1.05	8.80
Group 4	1.1	7.78	1.29	0.19	0.987	0.002	0.96	7.50
Group 5	−1.1	8.85	−1.14	0.15	−0.987	0.002	−1.00	5.30
Group 6	−1.2	5.93	−1.23	0.19	−1.19	0.002	−1.20	8.60

## Data Availability

Data are contained within the article.
